# Holding space and transitional space: stroke survivors’ lived experience of being on an acute stroke unit. A hermeneutic phenomenological study

**DOI:** 10.1111/scs.12824

**Published:** 2020-02-17

**Authors:** Kitty M. Suddick, Vinette Cross, Pirjo Vuoskoski, Graham Stew, Kathleen T. Galvin

**Affiliations:** ^1^ School of Health Sciences University of Brighton Eastbourne UK; ^2^ Centre for Health and Social Care Improvement School of Health and Wellbeing University of Wolverhampton Wolverhampton UK; ^3^ Present address: Faculty of Sport and Health Sciences University of Jyväskylä Jyväskylä Finland

**Keywords:** acute stroke unit, hermeneutics, holding, lifeworld, lived experience, phenomenology, spatiality, stroke survivors, transition

## Abstract

Despite substantial reorganisation of stroke unit provision in the United Kingdom, limited qualitative research has explored how stroke survivors experience the acute stroke unit. This hermeneutic phenomenological study used accounts from four stroke survivors who experienced one of two acute stroke units. Through detailed analysis, the acute stroke unit emerged as a meaningful space, in two distinct but interconnected forms. As *holding space*, the unit was understood to offer protection and safe haven, as the stroke survivors looked to cope and respond to the temporal, bodily, biographical disruption and significant vulnerability brought about by stroke and by being in hospital. *Holding* was fulfilled by different people (including their fellow stroke survivors) and reflected a human response to human need and existential vulnerability. This space, and the practices within it, functioned to hold them intimately but also at a distance from their prestroke lifeworld. As such, the acute stroke unit *holding space* was intertwined with how it supported, encouraged or provoked transition. In the *transitional space* of the acute stroke unit, stroke survivors described how they survived the hospital‐healthcare space, stroke unit and poststroke space. This paper articulates how transition was meaningfully signified through its absence or presence, as they transformed, relinquished or re‐asserted their ‘self’, and in one case, recovered whilst ‘in there’. The findings of this study provide phenomenological insight into stroke survivors’ lived experience, the meaningful holding and transitional contribution of the unit, and how these spatial forms were intertwined. These insights are discussed in relation to the existing evidence base and stroke unit provision.

## Introduction

Recently in the United Kingdom (UK), traditional stroke unit services were closed or restructured into hyperacute^1^ and/or acute stroke units^2^. An acute stroke unit has been described as ‘one that treats patients usually in an intensive model of care with continuous monitoring and nurse staffing levels’ (1, p8). The shaping and auditing of these services have emphasised medicalisation, technicalisation and objectification. Although important, this can obscure the human dimensions of healthcare 2 and that which is less visible 3 and/or quantifiable.

To date, qualitative research has explored individual aspects of stroke unit experience, such as being admitted to a mixed‐sex environment 4, the meaning of rehabilitation 5, self‐regulation and transfer anxiety 6, hope 7, 8 and goal setting 9. Experiences of acute stroke unit provision have also been partially captured within a broader context, such as acute hospital care 10, organised stroke unit provision (including hyperacute, acute, rehabilitation and combined stroke units) 11, or a stroke hospital pathway 12. However, limited work has been undertaken in the UK and describing the type of service is often overlooked.

Despite these limitations, research suggests that experiencing an acute stroke unit or a similar type of context, or aspect of it, can be confusing 13, involve disassociation, uncertainty, fear, distress, anxiety, suffering, appreciation (regarding life and care received), reappraisal, and a significant need to make sense and search for understanding and information 6, 11, 14, 15. Hope 7, 8, physiotherapy and instances of recovery have also emerged as meaningful 6, 13. Although rehabilitation has been reported as significant in the acute stroke unit, research indicates that it can be limited or missing 5. Swedish and UK stroke survivors described striving for independence (work and practice), accepting support, and/or experiencing passivity within the hospital and stroke unit setting, respectively 6, 15. Specialist staff, technical equipment and an appreciation of relationships, staff attitudes and commitment may also meaningfully contribute to patients’ experience 4, 11, 12. However, stroke survivors also articulate limited time, staffing and workload and resource issues 11, 15, 16 within their experience of acute stroke settings. The most recent research 11, has indicated that these and other factors (such as targets and pathways) in organised stroke unit provision, can challenge stroke survivors’ and health professionals’ capacity to build relationships and thereby the practitioners’ ability to provide reassurance, information or address holistic needs.

Since the reorganisation, there has been insufficient dialogue about the meaningful components of acute stroke unit care. Research has yet to explore the experience *phenomenologically*, in its’ entirety, and give voice to those with direct experience. This study looked to attend to the subjective, lifeworld of stroke survivors and their meaningful, practical concerns to provide much needed insight and understanding for those working in acute stroke unit settings.

## Methodological perspective

This study adopted a hermeneutic phenomenological methodology to gain access to a phenomenological world of meaning, embodied with practical concerns and dealings, encountered and embedded within human everyday living 17, 18. The idiographic nature of hermeneutic phenomenology holds particular resonance for healthcare and stroke provision, both humanly sensitive, complex practices involving multiple, varied individuals with their own history, context, beliefs, understanding and meaning making. Congruent with this, the development of hermeneutic understanding was directed towards the individual, played out within their story and their related *texts* 19, 20 *,* to deepen what is known about the acute stroke unit experience.

To empirically explore the lived experience, the researcher relinquished the natural (prereflective) everyday attitude and adopted a basic level phenomenological attitude (a special reflective act of consciousness). This involved attending to the intentional relation between the acts (noesis) and objects of consciousness (noema)^3^, 21; and how they and the phenomenon showed itself 22. Congruent with philosophical hermeneutics, language, dialogue and the hermeneutic circle^4^, were considered the mediums through which the conscious and ‘given’ as well as that which may not be as fully formed in thought, or as evident to the ‘utterer’ in their prereflective, natural attitude, were accessible 23. This methodology adopted the position that hermeneutics is our way of understanding and constituting our being‐in‐the world 17, 23. It is our interaction and dialogue with another and the text that initiates entering the hermeneutic circle 20. By sensitively attending to part and whole in the hermeneutic circle, the researcher begins to move from her horizon of lived meanings and prejudgements 23, towards a comprehensive, unified understanding of the horizon of another (fusion of horizons) 20. This involves opening up a co‐constituting dialogue and intersubjective space *between* the researcher and each person during the research interview 24. This dialogue carries on beyond the interview, reflecting back on itself, towards the phenomenon of interest (as part of the hermeneutic work). This indicates the hermeneutic and reflective process the researcher undertakes when working with the interviews and transcripts from each persons’ interview. Closely attending to what is said, not said, how it is said, the parts and the whole, what is brought to consciousness about the phenomenon of interest, in a cyclical, searching and interrogative way.

It is the quality of descriptions gathered about the phenomenon of interest; for nuanced depth of understanding; rather than the number of participants that takes precedence in phenomenological inquiry. Small sample sizes, from the use of single cases 25, to three to six participants 26, 27, 28, are appropriate 24, 29. In this study, four was selected to corresponded with Wertz 30 and Giorgi’s 26 proposition for at least three, to provide idiographic and typical variations from the personal accounts of those involved.

### Participants and setting

Stroke survivors had to have been admitted to one of the units in the two hospitals involved (Table 1) and be able to participate in the interview. The first four people who contacted the researcher and met the criteria were recruited. Two had experienced acute stroke unit 1, and two acute stroke unit 2. Three of them were in their 70s, one in their 50s, and their time lapse since stroke ranged from three to twenty‐one months^5^.

**Table 1:** Description of the characteristics of the acute stroke unit setting

Descriptors	Acute stroke unit 1	Acute stroke unit 2
Approximate number of beds	20	20
Seven‐day therapy service	No	No
Early supported discharge service^a^	No	No
5 key characteristics of an acute stroke unit (developed by the Stroke Unit Trialists’ Collaboration (SUTC) (53, p, 7)		
Consultant with responsibility for stroke	No	No
Formal links with patient/ carer organisations	Yes	Yes
Multidisciplinary (MDT) meetings at least once a week to organise and plan patient care	Yes	Yes
Provision of information to patients about stroke	Yes	Yes
Funding for external courses and uptake.	Yes	Yes

^a^ A team offering rehabilitation in the community that replicates stroke unit care; this enables earlier home discharge than would be possible if the team was not available.

### Data gathering

Interviews were undertaken in participants’ homes and focused on gathering rich descriptions of experience. Participants were free to talk and expand without interrupting. The researcher would revisit points and comments made using prompts and probes. Combined, the data collected reflected 240 minutes of audio recordings, 95 pages of interview transcripts, as well as the researchers’ field notes and reflections. Although the length of the interviews varied with each participant, this did not detract from the meaning embedded in each narrative, and the depth evident in the corpus as a whole.

### Analysis

This was an inter‐relational activity and ongoing co‐constituting dialogue between the researcher, the participants/s, their associated text/s and the phenomenon 31, 32, that began with the interview and continued beyond, as the researcher undertook detailed analysis. This involved reading the transcripts multiple times, listening to audio recordings, noting interpretations and exploratory comments (descriptive, linguistic and conceptual 27 as well as embodied and sensed). The researcher (*KS*) attended to part and whole on multiple levels (the single word in the sentence, the extract within the whole text, the text within the complete oeuvre and the single episode within the complete life of the person) 33, and later across the ‘collective’ whole. This was operationalised by the organisation of preliminary themes, reflection upon the researchers’ horizon of understanding (fore‐understanding and prejudices 20, Table 2), and the *texts* alterity^6^. Reading, re‐reading, dwelling and ‘playing’ with themes, deconstructed sections, and all forms of text were utilised to explore spaces, clusters, patterns and connections for preliminary themes across the participants. These were developed through writing and returning to the individual participants’ analyses to test and interrogate that which related to the ‘collective’ whole (Table 3). None of the understanding developed was brought a priori, but emerged through detailed analysis, dialogue and interrogation of the accounts and associated texts.

**Table 2:** A brief summary of how understanding developed during the analysis process

Articulating how the researchers’ understanding developed
At the beginning of the study my pre‐understandings about the experience of being on an acute stroke unit reflected a preoccupation with both rehabilitation (through my physiotherapist’s lens) and caring. Due to my own experience in practice, I assumed services would be negatively experienced by stroke survivors, that the practitioners on the acute stroke unit would most likely have cut themselves off from caring, and that I would discover what was missing and in opposition (i.e. practice versus need, rhetoric versus reality). However, as I undertook the analysis, it became apparent that for most, rehabilitation was not meaningfully signified. And, although caring was important, it was in fact holding, and transition; both of which represented a more holistic, expansive and complex understanding; that emerged as meaningful to these individuals’ acute stroke unit experience. As I undertook Sarah’s analysis, space began to form (albeit undeveloped) within my thinking and understanding in various ways: the space between her lifeworld before and since the stroke, space in relation to absence and presence (on the stroke unit, and after leaving), and as I progressed further with her analysis; the acute stroke unit (and within it the hospital bay), that she experienced as a populated and practiced place. Jane’s account offered emergent insight that meaningfully signified the acute stroke unit as place, the relevance of being ‘in there’, how the stroke unit seemed to hold her at a distance from her pre‐stroke lifeworld, how this was interconnected with her self, feeling held in place, but then confined. Andrew’s analysis pointed towards the stroke unit (and in particular the significance of the nurses who practiced there) as different and special, standing apart and protecting him from the other, dehumanising wards of the hospital. As I continued my hermeneutic work, I began to understand how the nurses were meaningfully signified within Sally’s narrative, but in contrast, through her disappointment in their absence and limited proximity. The acute stroke unit thereby began to emerge as a space of meaning; a lived space that was peopled and practiced with others and interwoven with the lifeworld of the stroke survivors involved, and other meaningful spaces.

**Table 3:** Example extracts, themes developed in the individual analyses, and their contribution to the final, comprehensive understanding of how the acute stroke unit was meaningful experienced across the ‘collective’ whole

Extracts	Preliminary themes and *subthemes* from the individual analyses	Themes contributing to comprehensive understanding for the ‘collective’ whole
‘Well I can’t use the word comfortable….I wasn’t comfortable at all, but because I knew that I was safe there I was able to sit and do that, where I think if you’re not safe…you’re anxious all the time and I probably wasn’t as anxious…I was anxious to get out because I always am, and they just laughed because they knew me from before.’ (Jane)	An interaction between place and self *Place for sensing/thinking* *Familiarity of place* *Feeling conflicted and confined within place (Jane)*	Holding space offering protection and safe haven
‘The nurses were all so busy…none of them had time to sit down and talk to you, you know’ (Sally) ‘Um… I don’t know really. I suppose when they’re trained, you know, you go in another ward, you get on with it, yeah… But not there.’ ‘I suppose like um… Macmillan Nurses, you know they’re trained to do that, yeah.’ (Andrew)	A potentially less significant acute stroke unit *Busyness and absence of nurses (Sally)* *The nurses as significant for coping with vulnerability experienced in the acute stroke unit after stroke (Andrew)*	Holding through the spatial practices of nurses
‘and one day she was very, very ill, she started to be sick and obviously very poorly and I rang the bell and sort of asked….when they were seeing to her I said don’t close the curtains around her, I want to watch her, and I said I’ll let you know if she needs anything else. We were all afraid that she would choke, you know, when she was being sick’ (Sarah)	Responding and transitioning through a social process of rebuilding within a community of strangers *The communal body of the hospital bay (Sarah)*	Holding through the spatial practices of others‐ including the spatial practices of fellow stroke survivors
‘oh god a lot. Because you have a lot of time in there. You know…there’s not..not much time that I sit’ ‘it’s just things like a new life‐ so you’re going to change your life’ (Jane) ‘you just have to learn patience in hospital, you know, things can’t happen the minute you want, they do the best they can, but you know, sometimes you have to be prepared to wait’ ‘you have to be realistic and accept’ (Sarah).	An interaction between place and self *Being situated in place‐ acute stroke unit as place* *Place and time relationship* *Place (space) for sensing/ thinking (Jane)* Complex process of transition of self and making sense in response to disruption *Adopting a hospital persona (protection from disruption) (Sarah)*	Transitioning self: surviving the lived space
‘so I more or less said right, well what’s next? What do we do next?… I felt whatever it was I was told to do or to expect, I could get on with it’ ‘I can also motivate myself to do more. And I think that’s important, I think some people give in so easily and just think oh that’s’ it’ (Sarah) ‘once the make‐up goes on and the lipstick goes on they’re thinking ‘oh my god’..’…’She’s on her way, look, and saying please let me just go out for a while, let me have a shower this morning. I was a pain in the neck really. And they just let me get on with the things that they know that…you know, they do an amazing job they really do’ (Jane)	Complex process of transition of self and making sense in response to disruption *Biographical confirmation and reconstitution‐ re‐establishing continuity (Sarah)* An interaction between place and self *Feeling confined and confined within place* *(Jane)*	Re‐emergence of self
‘I think it was more intense there. They really did work very intensely and there were always two of them, you know, so one to guide,’ ‘they had me…they would throw a ball and have me trying to catch it, and kick a ball, you know, which I could hardly move at all,’ ‘it meant you were on the road to recovery’ (Sarah).	Responding and transitioning through a social process of rebuilding within a community of strangers *The physiotherapy contribution (Sarah)*	Transitional space for recovery

### Findings

For these four stroke survivors, the acute stroke unit was experienced as a lived space^7^ in two differentiated but intertwined forms: holding space and transitional space.

### Holding space: offering protection and safe haven

The acute stroke unit experienced as holding space was a meaningful place that stood apart; offering protection and safe haven as the stroke survivors dealt with their poststroke space; an abstract, frightening, uncertain space, awash with vulnerability and disruption; as well as the hospital‐healthcare space. Andrew recounted a series of processes and events in detail; what seemed to be an efficient processing by multiple agents, as he arrived at hospital and the Accident and Emergency department. He described how he was given an injection that was ‘meant to save you’, but had a nosebleed, and it was stopped. He explained:

‘so that was it‐ I mean‐ I went up on the ward’.

Andrew needed the safety and protection offered by the acute stroke unit following this abrupt, matter of fact but tragic, end to the saving. Feeling safe and protected were also meaningful to all four stroke survivors as they found themselves confronted by their own, and others’ significant vulnerability, biographical disruption, corporeal fragility and helplessness after the stroke:

‘… when it first happens it does come as a big shock, you know, especially when you want to get out of the bed to go and get washed and stuff and you’ve got to ask somebody’ (Sally).

This holding space held them intimately, but also at a distance from their prestroke lifeworld, and place of home so that they could start to think about what had happened, respond to the stroke, and in some cases look to the future. For Andrew, this holding space was also distinct from the rest of the hospital, thereby providing protection by way of ‘space between’:

‘…it seems different to any other ward, you know, they [nurses] seem they’ve got time for you. Yeah it was lovely’ (Andrew).

### Holding through the spatial practices of nurses

The nurses’ spatial practices (through their presence, kind and caring ministrations) helped the stroke survivors cope with the sudden, significant disruption evident after their stroke.

‘She was really kind, she’s the nurse that gave me a wash the first day I woke…, in the morning, that devastated me that… someone having to wash me, um… that was horrible’

‘… I remember her telling me that she lived in a caravan and because she worked so hard and they didn’t get that much money and she couldn’t afford to live in a house, so she lived in a caravan, poor thing.’ ‘And I remember that, I always remember that’ (Jane).

There was a suggestion that this nurse was tacitly respecting and acknowledging Jane’s vulnerability. ‘Holding’ in a sense to distract, connect, and/or offer up her own vulnerability, as part of her spatial practice, to make the personal care more bearable. Thus, the stroke survivors’ experience of holding, through the nurses’ spatial practices (kindness and sensitivity), was understood to help them cope and in some cases respond to the disruption brought about by the stroke:

‘I couldn’t praise any more, them any higher, because they were just so kind and so nice, and because I was comfortable I was able to sit…and think about things’ (Jane).

For Sarah as well as Jane, the nurses’ holding meant that they felt remembered and ‘known’; maintaining their sense of self after the stroke and within the space of the unit. Meaningfully related to the previous theme, Andrew’s experience of the nurses’ holding, meant he did not give up and was protected from additional hurt and vulnerability (being in a different ward, dehumanised or alone). This holding space, practiced by nurses, was thereby thought to be a nurturing and caring space. However, spatial practices surrounding or within it could also contribute to disruption and vulnerability. For all of the stroke survivors, this engendered feelings of diminished control and powerlessness:

‘Well no, they actually… you know when you’re getting nothing found [possibly tests, possibly improvements], and they tell you, “you’re going home” and that’s it’ (Andrew).

Although the nurses were understood to provide a kind, loving, caring, helping presence, normality, practicality, protection and physical assistance, holding was not without issues. For Sally, the nurses’ busyness meant she did not feel sufficiently held. For Sarah, the supportive and protective holding space during the day seemed to contravene human need, becoming a lonely, fearful space at night:

‘We dreaded nights because we knew there was not going to be anywhere near enough staff on duty and we all used to say to each other, “try not to need anybody, because you won’t get anybody!” [laughs]’ (Sarah).

Her account provided access to a less overtly expressed, but significant sense of disappointment and concern for safety, with vulnerable people left to cope and suffer on their own.

### Holding through the spatial practices of others including the spatial practices of fellow stroke survivors

Sarah, Jane and Sally referred to a more general and broader ‘they’ of the acute stroke unit. Although this indicated that holding was not the exclusive domain of nurses, they provided no further insight other than a commitment to do ‘as much as they possibly can for you’, and not ‘just do the basics’ (both Jane).

Within Sarah’s analysis, the hospital bay emerged as a meaningful space that *she* and her fellow stroke survivors, populated and practiced. These stroke survivors were disrupted from their at‐home‐ness, within their body, lifeworld and biography, to stay in the foreign and separate (but holding) stroke unit space. To compensate, Sarah and her fellow stroke survivors uniquely forged a temporary community. Sarah described how they shared skills, abilities, knowledge, encouragement, support and responsibility for each other in this space. How they *held each other*; compensating for the disruption caused by their stroke as well as the nursing situation:

‘because always, if one of us needed something and couldn’t do it, one of the others would… if anybody could move and I’d perhaps dropped something… if somebody was mobile, they would come and pick up whatever I’d dropped. So we helped each other out wherever we could, because again, it’s just so understaffed and especially at night.’ (Sarah).

### Transitioning self: surviving the lived space

Three of the stroke survivors described transitions orientated around the self that helped them survive the lived space of the acute stroke unit, their poststroke space, and/or the hospital‐healthcare space. Andrew’s analysis signified how he relinquished the hypervigilance evident in his account of having the stroke and being in the Accident and Emergency department. He was understood to passively transition, sleep and recuperate, surrendering of himself, knowing he was *held*; safe and cared for by the nurses. This was how he survived the significant (albeit less explicitly articulated) vulnerability that pervaded his account.

Sarah’s experience of being on the acute stroke unit involved different transitions. To survive the acute stroke unit and hospital healthcare space, she temporarily transformed into a different way of ‘being’; by reducing her expectations, relinquishing control and being accepting. In doing so, she continued to feel agentive, and protected herself from the frustrations and disappointment she would otherwise feel.

Like Andrew, transition of self and how it related to being *held*, also emerged within Jane’s account. In the holding space of the acute stroke unit, Jane experienced a different way of being as she paused, was still, and reflected on how she was going to respond to the stroke. However, quickly and dynamically she began to feel held in a negative sense, as she looked to escape the units’ confines and do things for herself. The staff went beyond what was expected, but some of them varied in how responsive they were to her quickly changing need:

‘once the make‐up goes on and the lipstick goes on, and the perfume, they’re thinking oh my god’ (Jane).

This transition was therefore part constituted within Jane’s experience of surviving the lived space, but also meaningfully related to the next contributing theme: re‐emergence of self.

### Re‐emergence of self

Within the acute stroke unit, Jane and Sarah were understood to transition, reinstating their sense of self and agency in unique ways. Sarah’s reinsertion of ‘I’ within her narrative pointed towards this transformation. It was also meaningfully signified through its’ utility within her recovery (see final theme).

Jane’s re‐emergence of self, reflected her need to do anything asked of her, refuse, even bend the truth, to ‘get home’; instigating her escape from the holding space that now represented restriction and over‐protection:

‘and then she [occupational therapist]… she made me go up this… and I said “I’ve just gone up the stairs, I just want to get home” and she said… I will l never forget, she was going… “well I’m going past that way and I could go in your house and see”… and “I don’t want any”… She said “and we could see if we put rails up and that would be another five days”. And I went “no I don’t need any”. And I remember telling quite a few fibs and saying “oh um… I’ve got crutches, I’ve got things in my garage [laughing] someone can lend me this” and… just so I went home. I’m not… I’m horrid…. I’m very naughty really to do all that,’ (Jane).

### Transitional space for recovery

This theme reflected how Sarah described using her own, and other resources (the community of the hospital bay, nurses and physiotherapists), to assist her recovery, reduce the bodily, biographical and temporal disruption brought about by stroke, re‐establish her continuity, and give her a sense of hope for the future. Sarah explained how the nurses moved sensitively from holding to transitional practices:

‘initially of course they’d bring a bowl of water then gradually, they would bring a bowl and you’d sit in your chair and they’d wash you. But then they would say “now how much do you think you can do for yourself?”, and I would say “oh well I’ll have a go at what I can do”.’ (Sarah).

In contrast, there was no gradual transition within physiotherapy. It was immediate, challenging and intense and was perceived to play a significant part in Sarah’s recovery after stroke.

Other than a singular mention of physiotherapists by Jane; ‘they were just really nice and encouraging, you know’, and the occupational therapist already mentioned in the preceding theme; recovery and therapy staff were less evident (Jane), or completely absent (Andrew, Sally) in the other stroke survivors’ accounts.

## Discussion

This study has illuminated these stroke survivors’ experience of the acute stroke unit as a holding space. *Holding* offered variant meanings beyond caring and helping, and signified the human need of being and feeling held, being held close, held together, and held apart within the lived space of the acute stroke unit, and spatial dimension of the lifeworld. The findings illustrate that holding may be particularly meaningful in the acute stages after stroke as well as at certain times, that is when stroke survivors first experience being helped with self‐care, and at night.

Whilst on the unit, these four stroke survivors were understood to experience already highlighted existential concerns that related to their diminished control, as well as biographical disruption, a threatened sense of self, and an awareness of corporeal fragility. The majority of this disruption and loss of control were brought about by the stroke. However, all the stroke survivors felt the spatial practices within the hospital and acute stroke unit space, in different ways, could accentuate their sense of vulnerability, which perhaps related to how Andrew in particular, described his appreciation of *holding*. As Malone 36 has articulated:

‘Leaving behind the place of *home* and entering into the unfamiliar institutional spaces of the hospital, our understandings of intimacy and distance are challenged by intrusive examinations, loss of private space, the threatened foreshortening of our life horizons, and the need to depend upon the kindness of strangers as we seek not only wellness, but re‐emplacement in our bodies and our lives.’ (p, 42).

A hospital space that can engender vulnerability echoes with other work. The participants in Arntzen et al 37 and Yeung et al 38 explained how their inpatient experience seemed separate from reality, their lifeworld and family. Arntzen et al’s 37 stroke survivors felt safe and secure, whilst sometimes not feeling safe and secure in their contrasting experience of day and night. At the same time, some of these Norwegian stroke survivors felt the inpatient situation meant they thrived 37. All of these seemingly differing positions resonated with the variant meaning present within these four stroke survivors’ lived experience of being on an acute stroke unit.

Taken together, this paper proposes that ‘holding space’ is responsive to need, about the emotional environment 39, 40, and the safety, and nurturing qualities of the space produced. In contrast to the existing evidence base, these findings offer a more positive understanding regarding the stroke survivors’ experience of acute stroke unit staff members’ (and in particular, the nurses’) holding. This was evident through their efforts to maintain dignity, integrity of self and minimise vulnerability and was in contrast to other stroke survivors’ experiences of care in a number of settings and hospitals across the UK 10, as well as a recent meta‐ethnography 41.

These findings have illuminated that stroke survivors can experience too much or too little holding. This has emerged only partially elsewhere, with patients describing feeling stuck in a cage in hospital 42, imprisoned 14, 43, trapped 44 and missing contact with the nurses 15. What was significant in the present study, was that holding was understood as an essential part of the acute stroke unit experience, that the nurses’ ability to hold was influenced by deficiencies in staffing, and that ‘holding close’ and ‘holding apart’ (hence distance and proximity) were important for transition. Congruent with Ryan et al 11; building meaningful, relational and person‐centred practices in acute stroke care; can be challenged by a backdrop of staffing, resource, time pressures; targets, pathways, and technical, fast‐paced processing.

Holding however it was fulfilled, and by whom; was the necessary foundation, in some cases, the impetus for transition. It is proposed that this reflected the nurturing and distance needed to fulfil the role of ‘good enough mother’ 45, ^8^ for stroke survivors on‐their‐way‐to‐becoming. ‘Holding’; understood as nurturing refuge 46, functioned so that stroke survivors could ‘be’, and afforded them time and space to understand their current circumstances, experiment and ‘play’. The relevance of spatiality within narratives of migration 35, and nature as ‘containing and holding space’ for immigrant and refugee children 47 has emerged in other fields. Like with Hordyk et al’s 47 children, the *holding space* for stroke survivors’ was thought to offer security, trust and confidence, which meaningfully related to how they felt they could develop, re‐establish and reconstitute their self and agency, in varying degrees.

### The transitional space of the acute stroke unit and the relevance of self

Traditionally in the stroke field, transition has been emphasised as discrete time points, or events that reflect movement from one place to another, like returning home 48. More recently, two grounded theory studies explored transition and transformation, respectively 49, 50. This study adds experiential weight to transitions ranging from being absent (in one case), to present and in process (for the other three stroke survivors) during the acute phase of stroke. These were not limited to recovery, but included protective, necessitous and potentiality‐driven transitions. Complexity and multiplicity in a similar fashion to Timothy et al 49 were evident as; transition took variant forms, and different selves could co‐occur simultaneously (i.e. the co‐existence of Sarah’s temporary ‘hospital’ self and re‐emergence of self). These findings offer insight that a number of the elements articulated in the theories developed above (i.e. vulnerability, taking action, seeking control, examining and thinking about self and life, anchors for grounding, and the complexity, co‐existence and flux of divergence and cohesion) 49, 50, may be in process whilst stroke survivors are on the unit. As such, holding may be particularly valuable during this time.

What has emerged as especially illuminating was the individualised and dynamic ways the stroke survivors relinquished, accepted or re‐asserted control within their acute stroke unit experience and the hospital space, how this was intertwined with holding and transitional space, and how they and others fulfilled holding and facilitated transition. Some of these transitions (passive relinquishing of self) partially mirror that of other stroke survivors 15, 42. Norwegian stroke patients’ hospital (stroke centre) experience, included passively putting themselves in the staff’s charge, without demands and expectations, thereby becoming objects of depersonalised care 15. However, in contrast to Olofsson et al 15, it was through the nurses’ holding practices in this study, that Andrew in particular, and Sarah and Jane (through their own efforts and/or others’ holding), *did not* feel depersonalised.

Viewing the acute stroke unit as a lived space, in these different meaningful spatial forms can re‐orientate the focus of care towards holding and the emotional and psychological support of the individual, for their own development, and potential transition. This resonates with lifeworld‐led healthcare; an existential view of the person and wellbeing, and the complex and multi‐dimensional nature of transition:

‘Professionals led by lifeworld knowledge do not therefore just offer technical solutions, but are able to offer ‘paths’ for the patient to step into in their life’s journey’ (51 p, 270).

How the stroke survivors involved in this study *practiced* within the space of the acute stroke unit through their actions, behaviours, and responses in particular and dynamic ways, needs greater recognition as well as further exploration.

### Strengths and limitations

Some participants were describing their experience after a considerable delay. However, Halldórsdóttir 52 suggests that in the midst of experience a participant’s ability to reflect may be limited. It is nevertheless important to acknowledge that the stroke survivors’ thinking about their acute stroke unit experience would be shaped and viewed from their recollections, alongside what they felt they could share with the researcher, and will always be relative to the time of their admission, the UK, these specific hospitals, these units and individuals. Most importantly, it is necessary to acknowledge the findings as representative of the researchers’ interpretation of these peoples’ experience from their perspective at the time of interview. They are not proposed as an absolute truth, rather a rigorous, thorough, detailed, empathic and contextualised understanding of the acute stroke unit as lived through by these four individuals. Understanding, that this paper argues, could have direct relevance to the people involved (i.e. stroke survivors, relatives and health practitioners), and complement the existing quantitative, medicalised dialogue about acute stroke unit care.

## Conclusion

This study has taken place when there is still ‘space’ to explore acute stroke unit provision before the reorganisation and policy change moves us too far away from human experience, and the meaningful spaces lived through. These findings offer an opportunity to think and engage with the complex, meaningful experiential nature of being on the acute stroke unit from the stroke survivors’ perspective that can have specific, meaningful relevance to caring, humanly focused, healthcare practice.

This research has uncovered the differentiated, but intertwined holding and transitional spatial contribution of the acute stroke unit. Holding emerged as a more detailed and nuanced interpretation that reflected a human response to human need and existential vulnerability, that was practiced by different people (including nurses and stroke survivors), and was not only the essential foundation, but could also be the impetus for transition. This paper proposes that this meaningful part of the acute stroke unit experience is missing from contemporary definitions and conceptions of acute stroke unit care.

By venturing a re‐imagining of the space of health care as practiced by both stroke survivors and healthcare practitioners, this paper aims to enhance sensitivity and recognition of the different ways that patients’ practice, respond, transition, and thereby prompt further exploration of this topic. Through the development of understanding about the varied transitions emergent within the stroke survivors’ time on the acute stroke unit (those for protection, necessity and recovery), that they can co‐exist and that some stroke survivors can quickly and dynamically move between them, health practitioners can better understand the patients’ lived experience. It can therefore provide an opportunity to enhance their sensitivity and attunement, so that they can better recognise transition, understand transition, support transition, talk about transition and modify their holding accordingly.

## Author contributions

KS was responsible for the design of the study, data collection and analysis. VC, PV, and GS supervised the study. KS drafted the manuscript, which was developed in close collaboration with PV and KTG.

## Funding

The University of Brighton provided a small grant that supported the completion of the study. No grant from any funding agency was received. The authors declare no conflict of interest.

## Ethical approval

All procedures were in accordance with the ethical standards of the University of Brighton, Faculty of Health and Social Science Faculty Research Ethics and Governance Committee, the National Research Ethics Committee (09/H1107/111), and the 1964 Helsinki declaration and later amendments. Potential participants were provided with written information about the study and provided informed consent. They could withdraw at any time, all information was anonymised, pseudonyms were used, and data managed securely.

## Notes

^1^ Hyperacute stroke unit: providing care in the first 72 hours after stroke.

^2^ Acute stroke unit: normally providing care up to 7 days.

^3^ The intentional direction of consciousness towards the meaning (Noema‐ unities of meaning) and the manner in which this is given (Noesis: the acts of consciousness).

^4^ The hermeneutic circle is an ongoing circular movement between part and whole, as understanding becomes more complete 19.

^5^ The time period for when they were admitted as well as time since stroke was extended to assist recruitment.

^6^ Congruent with Gadamer’s (2004) proposition, that in the act of understanding, ‘a hermeneutically trained consciousness must be, from the start, sensitive to the text’s alterity’ (20, p, 171).

^7^ A lived space meaningfully experienced and constructed through peoples’ actions, behaviours, relationships and practical engagement with the world and living situations 34, 35.

^8^ This has emerged from the child development field, along with the concepts of holding environment and transitional phenomena 45. ‘Good enough mothering’ represents the interplay between nurturing and distance required from the mother, for the child’s development of self, engagement with the world beyond the mother and their own unique creative, experiential growth 45.
